# Effect of copper sulfate on the survival and growth performance of Caspian Sea kutum, *Rutilus frisii kutum*

**DOI:** 10.1186/2193-1801-2-498

**Published:** 2013-10-01

**Authors:** Esmail Gharedaashi, Hamed Nekoubin, Mohammad Reza Imanpoor, Vahid Taghizadeh

**Affiliations:** Department of Fishery, Faculty of Fisheries and Environment, Gorgan University of Agricultural Science and Natural Resources, Gorgan, Iran

**Keywords:** Copper sulfate, LC_50_, Growth, Body weight, *Rutilus frisii kutum*

## Abstract

The aim of present study was to determine the LC_50_/96 h value of copper sulfate and determine the growth performance of Caspian Sea kutum) *Rutilus frisii kutum* (fingerlings during 60-days sub-lethal copper (Cu). After acclimation period to two weeks, for determine the LC_50_/96 h value, total of 27 aquarium with a capacity of 60 L each stocked with 10 fishes an aquarium. Tunney four aquarium and 8 concentrations of (Cu) composed the 24 treatments while 3 other aquaria were used as control. For each treatment, three replications were conducted. And experiment to determine the growth performance fish were transferred into fiberglass aquaria of 200 L water capacity for growth trials. The treated fish were kept in the aquarium containing sub-lethal concentrations of Cu (0.11 and 0.23 mg L^-1^) and affected to grow for 60 days, while control fish were placed in metal free water. The results indicated that median lethal concentrations (LC_50_) of copper to Caspian Sea kutum for 96 h exposure was 2.310 ppm. The chronic sub-lethal water-borne Cu exposure to the fish exerted that fish had significantly decreased final body weight in comparison to control group. The copper sulfate also had significant negative effects on specific growth rate (SGR) and feed conversion efficiency (FCE) in comparison to those fed the control group. The feed conversion ratio (FCR) and condition factor (CF) were significantly increased in comparison with the control group (P < 0.05). Also survival rate in experimental treatments in comparison with control group, was significantly decreased (P < 0.05). The treated fish exhibited significantly lower body weight and length gains than that of control. The increments of fish weights, fork and total lengths, condition factor, feed intake and condition factor of treated and control fish varied significantly during study period. Hence, concluded that copper sulfate is toxic for Caspian sea kutum (*Rutilus frisii kutum*).

## Introduction

*Rutilus kutum*, the kutum, or Caspian kutum is a fish of the family Cyprinidae from brackish water habitats of the Caspian Sea and from its freshwater tributaries. It is typically a medium sized fish. The population seems to have collapsed due to over exploitation and marine pollution. Its flesh and roe is enjoyed as food, and highly prized in Gilan and Mazandaran provinces in Iran.

The tremendous increase in the use of heavy metals over the past few decades has inevitably resulted in an increased flux of metallic substances in the aquatic environment (Yang and Rose 2003). The metals are of special concern because of their diversified effects and the range of concentration stimulated toxic effect to the aquatic life forms. Industrial wastes constitute the major source of metal pollution in natural water (Livingstone 2001). Aquatic systems are exposed to a number of pollutants that are mainly released from effluents discharged from industries, sewage treatment plants and drainage from urban and agricultural areas. These pollutants cause serious damage to aquatic life (Karbassi et al. 2006; Masri et al. 2002). The rapid increase in human population has escalated the demand for quality food, like fish, in the world. To fulfill the food requirements, fish assume greater importance because, it contains high quality proteins, fats and minerals. Another feature of fish is its ability to convert raw materials into high quality proteins more efficiently than other terrestrial animals such as sheep, goats, cows etc. Fish is an indicator to measure the freshwater contamination by heavy metals because they occupy different trophic levels in an aquatic ecosystem.

LC_50_ is the biological index of 50% mortality in an exposed population. The 96-hour LC_50_ tests are conducted to measure the susceptibility and mortality potential of biota to particular toxic substances (Boudou and Ribeyre 1997). Higher LC_50_ values are less toxic because greater concentrations are required to produce 50% mortality in exposed animals.

Copper (Cu) is an essential metal for all organisms including fish. It has an important role in metabolism and its concentration is well regulated. However, Cu is one of the most toxic metals to fish and affects various blood parameters, growth, behavior, enzyme activity, and reproduction (Sorensen 1991; Roesijadi and Robinson 1994; Nussey et al. 1995). Metals are non-biodegradable and are considered as major environmental pollutants causing cytotoxic, mutagenic and carcinogenic effects in animals (More et al. 2003). Metals are unique among pollutants, which cause adverse health effects, in that they occur naturally and in many instances are ubiquitous in the environment. Heavy metals have long been recognized as serious pollutants of the aquatic environment. The accumulation of metals in an aquatic environment has direct impact on man and aquatic ecosystem.

Chronic exposure of fish to water-borne Cu, Cd or Zn has been shown to cause a variety of physiological and behavioral changes including loss of appetite, reduced growth, ionic loss and increased fish mortality (Scherer 1997). Heavy metal contamination usually causes depletion in feed utilization in fish and such disturbance may result in reduced fish metabolic rate and hence causing reduction in their growth (Javed 2005). Growth is a sensitive and reliable endpoint in chronic toxicological investigations (Boeck et al. 1997). The present work was designed to investigate acute toxicity of copper sulfate and toxic effect of copper sulfate on the growth performance of kutum under chronic sub-lethal concentration to evaluate its potential to growth in contaminated water.

## Materials and methods

Juvenile Caspian sea kutum selected for this study were obtained from the fish seed hatchery in Gorgan, Iran. Caspian Sea kutum measuring 8 ± 0.5 cm in length and weighing 4 ± 0.5 gr were used for the experiment. They were brought to the laboratory and acclimatized for 14 days and the fish were fed with commercial pelleted feed during this period (Table 1).

**Table 1:** **Nutrient composition of experimental diets (%)**

Ingredients	%
Protein	54
Lipid	18
Fiber	1.5
Ash	10
Vitamin	2

Physico-chemical parameters viz. temperature, pH, total hardness, dissolved oxygen, total NH_3_, Na, K and CO_2_ of the treated and control media were monitored on daily basis by following the methods of APHA (1998). However, water temperature (24 ± 1°C), pH (7–7.5), dissolved oxygen (7.65 ± 0.55 mg^-1^) and hardness (275 ± 2.5 mg^-1^) was kept constant throughout the study.

### LC_50_ determination

Firstly, for investigate acute toxicity of copper sulfate all aquariums (60 L) capacity were filled with 50 L of dechlorinated tap water. A total of 27 aquariums that each stocked with 10 fishes were used in our experiments. Stock solutions of copper sulfate were prepared by dissolving analytical grade copper sulfate (CuSO_4_.5H_2_O from Merck) in double distilled water. Thirty fishes were used per concentration of copper sulfate.

Ninety-six hours acute bioassays were performed following in general OECD guidelines for fish acute bioassays (guideline OECD203, 92/69/EC, method C1) (OECD 1993). For determination of the LC_50_/96 h (lethal concentration) values, following a range finding test, eight copper sulfate (0.5, 0.75, 1, 1.5, 2, 2.5, 3 and 3.5 mg/L) concentrations were chosen for Caspian sea kutum. copper sulfate solutions were prepared by dilution of a stock solution with dechlorinated tap water. A control with dechlorinated tap water only was also used. The number of dead fish was counted every 12 h and removed immediately from the aquaria. The mortality rate was determined at the end of 24, 48, 72 and 96 h. During the toxicity test, the fishes were not fed. Acute toxicity test was conducted in accordance with standard methods (APHA 1998). LC_50_ values were calculated from the data obtained in acute toxicity bioassays, by Finney’s probit analysis (Finney 1971) using SPSS computer statistical software. With this method, the LC_50_ value is derived by fitting a regression equation arithmetically.

### Growth performance

Thereafter, to investigate toxic effect of copper sulfate on the growth performance of kutum under chronic sub-lethal concentration, This experiment was conducted in a completely randomized design with 3 treatments (two concentration of copper sulfate and a control), and three replicates per treatment for a total of six fiberglass tanks (each with a capacity of 200 L) 60 fishes were used per concentration of copper. Separate groups of 60 fish each served as control for copper. 5% and 10% of LC_50_/96 h concentration for copper sulfate (0.11 and 0.23 mg L^-1^) was used as sub-lethal level for kutum. In control experimented set up, no copper sulfate was added in water. Throughout the experimental period of 60 days, fish were fed with 4 percent body weight percent of their body weight (3 times a day) with the feed of digestible energy 2.90 kcal g^-1^ and 35% digestible protein. The treated fish were kept in the aquaria containing sub lethal concentration of copper sulfate and grown for 60 days, while control fish were placed in dechloridation top water.

The fish were weighed individually at the beginning and at the end of the experiment. In the termination of experiment, all fish from each tank were sampled and the final weight and length of body were measured. Growth parameters of fish were calculated based on the data of biometry of kutum fish. One-way ANOVA and Duncan’s multiple range tests were used to analyze the significance of the difference among the means of treatments by using the SPSS program.

## Results

### LC_50_/96 h of copper sulfate for kutum

Acute toxicity of copper sulfate showed that mortality is directly proportional to the concentration of the copper sulfate while the percentage of mortality is virtually absent in control (Table 2).

**Table 2:** **Showing correlation between the copper sulfate concentration and the Cumulative mortality (96 h) of Kutum**

Concentration(mg l^-1^)	N	Cumulative mortality (96 h)
0.00	30	0
0.50	30	0
0.75	30	0
1.00	30	1
1.50	30	3
2.00	30	8
2.50	30	18
3.00	30	26
3.50	30	30

Table 2, shows the relation between the copper sulfate concentration and the mortality rate for 96 h of kutum. Results according to SPSS18 analysis showed that the median lethal concentration (LC_50_) of copper sulfate to kutum for 96 h of exposure is 2.310 ppm (Table 3). In this experiment the behavior of fish remarkably changed due to the treatment of copper sulfate when compared to the control. The various locomotary responses exhibited by fish due to sublethal concentrations of copper sulfate during initial stage of exposure included restlessness, erratic and fast swimming, abrupt change in position and direction, jumping and overall hyperactivity were noticed. The fish showed surfacing tendency throughout the experimental period. Physiological responses like rapid opercular movement and frequent gulping of air was observed during the initial stages of exposure after which it became occasional. Neurological symptoms like jerking movements, frightening and loss of balance were not observed in copper treated kutum.

**Table 3:** **Lethal concentration (LC1-99) of copper sulfate (24-96 h) for Kutum**

Point	Concentration(mg^-1^) (95% confidence limits)	
LC_1_	0.928	(0.522-1.201)
LC_5_	1.333	(1.028-1.545)
LC_10_	1.549	(1.294-1.733)
LC_15_	1.695	(1.471-1.862)
LC50	2.310	(2.165-2.463)
LC_85_	2.926	(2.745-3.176)
LC_90_	3.072	(2.873-3.355)
LC_95_	3.288	(3.058-3.623)
LC_99_	3.693	(3.401-4.131)

### Growth performance

The results clearly showed that the copper sulfate had harmful effects on the growth parameters on Kutum. The feeding and growth parameters of Kutum are presented in Table 4. The growth performance and survival rate in control treatment of Kutum resulted better than copper sulfate group (P < 0.05). Also, the two different treatments of copper sulfate were significantly different for any of growth parameters. That, among the tow different concentrations of copper sulfate to Kutum, the greatest effect appeared to be obtained in treatments T2 (concentration 0.23 mg L^-1^ of copper sulfate). This is particularly false for specific growth rate, where the highest was obtained in the experimental control group. The feed conversion ratio (FCR) in the experimental treatments was significantly increased in comparison with control group (P < 0.05).

**Table 4:** **Growth parameters and survival rate of kutum (** ***Rutilus frisii kutum*** **) in experimental treatments (trial 1–2) and control**

Treatments	Control	T1	T2
Growth Indices	Free of copper sulfate	0.11 mg L^-1^copper sulfate	0.23 mg L^-1^copper sulfate
Initial weight (g)	3.90 ± 0.11	3.87 ± 0.04	3.91 ± 0.18
Final body weight (g)	4.74 ± 0.11^a^	4.62 ± 0.06^ab^	4.42 ± 0.17^b^
Body weight increased (g)	0.84 ± 0.01^a^	0.75 ± 0.06^b^	0.52 ± 0.03^c^
Specific growth rate for weight (% BW day^-1^)	0.32 ± 0.01^a^	0.29 ± 0.03^ab^	0.20 ± 0.02^b^
Feed Conversion Ratio (%)	0.60 ± 0.01^a^	0.66 ± 0.06^a^	0.95 ± 0.08^b^
Condition Factor	0.82 ± 0.01	0.81 ± 0.01	0.79 ± 0.03
Survival rate (%)	96.18 ± 2.18^a^	89.28 ± 4.28^ab^	81.99 ± 5.71^b^

Groups with different alphabetic superscripts differ significantly at P < 0.05 (ANOVA).

The treated fish exhibited significantly lower weights than control fish. The control fish also exhibited significantly better condition factor and feed conversion ratios than treated fish. The increase in weights and total lengths, condition factor of both treated and control fish varied significantly during study period. Copper sulfate exposure to fish resulted in increased oxygen and greater ammonia excretion by the fish.

## Discussion

Metal concentrations in aquatic organisms appear to be of several magnitudes higher than concentrations present in the ecosystem (Laws 2000) and this is attributed to bioaccumulation, whereby metal ions are taken up from the environment by the organism and accumulated in various organs and tissues. Metals also become increasingly concentrated at higher trophic levels, possibly due to food-chain magnification (Wyn et al. 2007).

The present study was initiated to find the susceptibility of the kutum to potentially hazardous copper sulfate on the survival and growth performance. The results showed that median lethal concentration (LC_50_) of copper sulfate to kutum for 96 h of exposure was 2.310 ppm.

The median lethal concentration 96 h (LC_50_) value of copper sulfate in other aquatic organisms: the LC_50_ for R. sumatrana, for 24, 48, 72 and 96 hours for Cu were 54.2, 30.3, 18.9 and 5.6 μg/L and For *P. reticulata*, LC_50_ for 24, 48, 72 and 96 hours for Cu were 348.9, 145.4, 61.3 and 37.9 μg/L respectively (Shuhaimi-Othman et al. 2010), which were lower than present study. The 24 h- LC_50_ of Cu was reported as 1.17 mg/L for *P. reticulate* (Park and Heo 2009), which were lower than present study. Gomes et al. (2009) reported that with juvenile Brazilian indigenous fishes, curimata *Prochilodus vimboides* and piaucu *Leporinus macrocephalus*, 96 h- LC_50_ of copper were 0.047 and 0.090 mg/L, for *curimata and piauçu,* respectively (Gomes et al. 2009), which were lower than present study. This indicates that different organisms have different sensitivity to heavy metals.

The toxicity reported by other studies differs from this study probably due to different species used, aged, size of the organism, test methods and water quality such as water hardness, as this could affect toxicity (Hodson et al. 1982; McCahon and Pascoe 1988). Toxicity of metals may vary depending upon their permeability and detoxification mechanisms (Darmono et al. 1990). And toxic effect of Cu on the growth performance of Kutum under chronic sub-lethal concentration showed that the copper sulfate had harmful effects on the growth parameters on Kutum.

These results are in accordance with the findings of Kim and Kang that reported the reduced growth rate of rockfish (*Sebartes schlegeli*) due to Cu stress and there was an inverse relationship between growth and Cu exposure (Kim and Kang 2004). Ali et al. (2003) observed reduced growth of *Oreochromis niloticus* under different (0, 0.5, 0.3, & 0.5 ppm) water-borne Cu levels (Ali et al. 2003). Also these results are in accordance with the findings of Mohanty et al. (2009) that determined the effect of copper on survival, growth and feed intake of Indian major carp, C mrigala for 60 days. They observed that feed intake in fish reduced significantly (P < 0.001) at all the Cu treatments. Significant difference was recorded in feed conversion ratios of treated (0.65) and control (0.55) fish. The control fish exhibited significantly better (0.26) feed conversion ratios than the treated fish (0.50) (Mohanty et al. 2009). Javed observed low feed conversion ratios in major carps (C. catla, L. rohita & C. mrigala) due to exposure of these fish to water-borne zinc (Javed 2005).

## Conclusion

In summary, under the indicated trial conditions, during our research, the present study was initiated to find the susceptibility of the kutum to potentially hazardous copper sulfate on the survival and growth performance. The results showed that median lethal concentration (LC_50_) of copper sulfate to kutum for 96 h of exposure was 2.310 ppm, chronic sub-lethal Cu exposure to the fish, kutum exerted significant impact on its weights, feed conversion ratio (FCR), condition factor and feed conversion ratios. The results of these studies may provide guidance to selection of acute toxicity to be considered in the field of biomonitoring efforts designed to detect the bioavailability of copper sulfate and early warning indicators of this heavy metal toxicity in Caspian sea kutum.
